# Improved quality and quantity of written feedback is associated with a structured feedback proforma

**DOI:** 10.3352/jeehp.2012.9.10

**Published:** 2012-08-13

**Authors:** Philip M. Newton, Melisa J. Wallace, Judy McKimm

**Affiliations:** 1 Graduate Entry Medicine Programme, College of Medicine, Swansea University, Swansea, UK.; 2 Institute of Experimental and Molecular Medicine, School of Medicine, Cardiff University, Cardiff, UK.

**Keywords:** Feedback, Assessment, Evaluation, Project, Transferable skills

## Abstract

Facilitating the provision of detailed, deep and useful feedback is an important design feature of any educational programme. Here we evaluate feedback provided to medical students completing short transferable skills projects. Feedback quantity and depth were evaluated before and after a simple intervention to change the structure of the feedback-provision form from a blank free-text feedback form to a structured proforma that asked a pair of short questions for each of the six domains being assessed. Each pair of questions consisted of asking the marker 'what was done well?' and 'what changes would improve the assignment?' Changing the form was associated with a significant increase in the quantity of the feedback and in the amount and quality of feedback provided to students. We also observed that, for these double-marked projects, the marker designated as 'marker 1' consistently wrote more feedback than the marker designated 'marker 2'.

## INTRODUCTION

Feedback is an essential part of any educational assessment [1], acting as a means by which students can improve by identifying areas for change and highlighting positive elements that the student should take forward [2, 3]. The latter form of feedback is especially important for transferable skills projects, because it is anticipated that students will be developing skills for long-term use in their professional careers, e.g., communication skills, literacy and data handling. Concerns about the quality and quantity of feedback provided to students have been a feature of the higher education literature for a very long time, but have been recently amplified by increases in student numbers and results from national and international surveys of student satisfaction [4]. For example, in the United Kingdom the 'National Student Survey' consistently identifies the provision of feedback as an area where higher (tertiary) education institutions can improve their service to students [5].

Interventions to improve the quantity and quality of feedback have included the use of recorded audio feedback, which appears to engage students more than written feedback and is more efficient [6] although it may be limited by resource and technological considerations [7]. Many feedback guides also suggest the use of structured feedback forms, although these can be cumbersome and the structure may be ignored by the teacher [8]. It is also not clear whether much research has been conducted to determine whether there is a real benefit to either learners or markers in using a structured proforma rather than a free-text method-a general search of the literature on feedback did not reveal any examples of such work in education, and neither did a specific search of the ERIC and PubMed databases for any of the terms "feedback form", "feedback proforma", "structured feedback", "blank feedback", and "free-text feedback".

There are also 'concerns about the concerns'. A review of the literature on assessment feedback in 2003 noted that concerns about feedback provision were based upon very few studies, conducted with limited sample sizes [9]. Thus there is a need to add to the evidence base by providing quantitative and qualitative analyses of feedback provided to students for a range of assignments. This evaluation addresses that need by considering the differences in both the amount and depth of feedback provided to students before and after an intervention to replace a free-text feedback form with a structured feedback form.

## METHODS

### Background and context

Data were analysed as part of the routine evaluation of educational processes at a medical school in a university in the United Kingdom. Additional consideration was made for the need to improve feedback following the results of the 2009 National Student Survey which highlighted giving feedback on assessments as one of the areas in which this institution (in common with most UK universities and programmes) performed most poorly. Students on the MBBCh Graduate Entry Medicine programme are required to complete a series of transferable skills projects, the aim of which is to develop some of the non-clinical (non-technical) skills that they require as practising doctors. These projects are part of the 'student selected component' (SSC), a requirement from the United Kingdom regulator of medical education, the General Medical Council, that at least 10% of undergraduate medical curricula should provide optional components (i.e., outside the core, mandatory curriculum) for students [10]. Within the SSCs therefore, students are free to choose any topic they like within a broad subject area (e.g., neuroscience). Students complete six projects in the first two years of the curriculum: 1) group project, 2) oral presentation, 3) patient information leaflet, 4) poster presentation, 5) literature review, and 6) free choice project in a format chosen by the student. All students complete each assignment type once. Assessments are summative and criterion-referenced with a pass mark of 50%. The criteria and marking rubric comprise six domains: content, understanding, sources, structure, style, and development of ideas. Each assessment is double-marked by two markers, designated as 'marker 1' or 'marker 2'. The grade awarded by marker 1 stands as that which is awarded unless there is a >10% difference between the marks allocated by examiners, in which case a third examiner (marker) is used. Both markers provide written feedback. Each assignment is completed by approximately 70 students, with 6-7 students assigned to each pair of markers. Thus there are approximately 10 pairs of first and second markers. Written feedback is provided, along with the mark, within three weeks of assignment submission.

### Evaluation conditions

Prior to the intervention, feedback had been entered by the markers into a free-text field of a Microsoft Access database, with a numerical mark assigned to each of the six assessment domains. The new intervention being evaluated here involved a change to the conditions under which structured feedback was provided. Markers were still required to assign a numerical mark to each of the six domains, but also to complete two feedback-entry fields for each domain: one entitled 'what was done well?' and one entitled 'what changes would improve the assignment?', thus breaking the single free-text field into 12 smaller fields. The design of these questions was based upon a need to facilitate a range of different feedback types without being prescriptive [1]. Assignments submitted in 2009/10 were given free-text feedback and assignments submitted by a different cohort of students in 2010/2011 were given structured feedback. The same types of assignments were evaluated for each cohort (see below), although they were marked by slightly different groups of markers (see results and discussion). Markers were not notified about any changes to the feedback form nor given any additional instructions. They were not aware that an evaluation of their assessment would take place.

The evaluation measures compared for the free-text and structured feedback forms were as follows:

1. The average amount of feedback provided per student (number of words);
2. The average amount of feedback provided per student (number of statements);
3. A breakdown of the average number of statements provided by marker 1 and marker 2; and
4. The depth of feedback provided, evaluated using a modification of Brown and Glover's methodology (Table 1).

### Word counts

The mean number of words provided as feedback was calculated by simple determination of the number of words written per marker, using Microsoft Excel.

### Coding of the feedback into 'Statement Type'

Numerous tools exist for the evaluation and categorisation of feedback. Here we use a tool developed by Glover and Brown [11] and Brown et al. [12]. It is highly relevant to the evaluation being undertaken here, having been developed for use in bioscience assignments and being simple, and thus transferable to the widely varying student-selected assignment topics for which we provide feedback. The tool has been used repeatedly for feedback evaluation (e.g., [7, 13-15] and has been adapted by the UK Open University for use as a publicly available feedback-coding tool (http://www.open.ac.uk/fast/pdfs/feedbck_codes.pdf).

The Glover and Brown tool classifies feedback statements based upon the depth of those statements. Feedback types are classified into category 1 (problem identified), 2 (problem identified and correct answer given), and 3 (problem identified, correct answer and explanation given) [11]. These are the N1, N2, and N3 categories shown here in Table 1. Glover and Brown [11] also suggest that positive feedback could be categorised in a similar fashion, thus equivalent to the P1, P2, and P3 shown in Table 1. Here we have added an additional category, 'X', meaning neutral feedback which is neither positive nor negative, is typically descriptive, and, crucially, does not identify useful advice for a student going forward.

The assignments under evaluation here are designed to develop transferable skills. Thus an essential role for any feedback provided is to allow the student to identify 1) things that they have done well and should continue, but perhaps most importantly, 2) areas for improvement. Coding of feedback into statements and classification by type was performed by two of the authors (PMN for the oral presentation assignment, MJW for the poster presentation). The guiding principle for all coding was 'if the student had to repeat the assignment, would the feedback be useful?'. Thus 'N1' and 'X' would not be useful. 'P1' and 'P2' would allow the student to identify those elements of the assignment that they should continue to include in the future, while 'N2' would identify those elements that they should change for the future. 'N3' and 'P3' are the 'deepest' feedback types, identifying and explaining areas of strength/weakness and also how these could be improved.

### Statistical analyses

All statistical analyses were made using Graphpad Prism ver. 5.3 (Graphpad Software Inc., San Diego, CA, USA). Details of the individual tests applied are described in the appropriate section of the results.

## RESULTS

### Initial evaluation

**Fig. 1:**
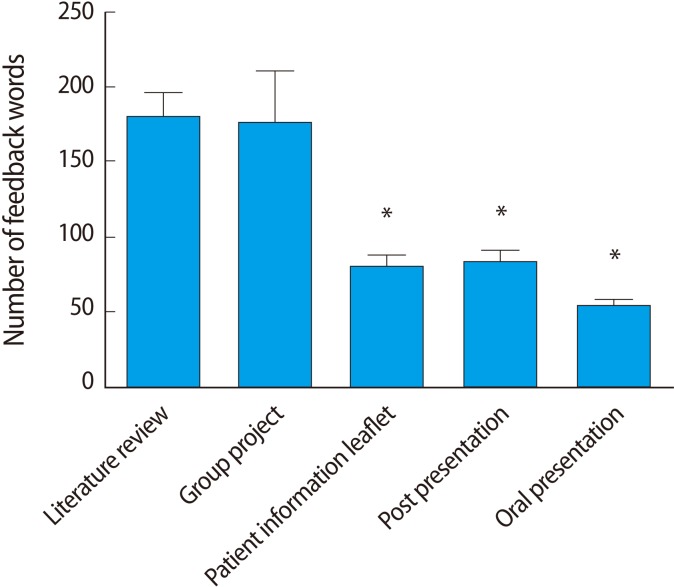
The mean amount of feedback returned for each transferable skills project. Data were compared by Kruskal-Wallis test with *post hoc* Dunn's multiple comparison tests. Patient information leaflet, poster presentation, and oral presentation projects received statistically less feedback when compared to both of the other projects. ^*^P<0.05.

Students complete six projects in the first two years of the curriculum: 1) group project, 2) oral presentation, 3) patient information leaflet, 4) poster presentation, 5) literature review, and 6) a project in a format chosen by the student. The total number of words provided in the feedback for each of assignments 1-5 was calculated. Feedback amounts were not calculated for assignment #6 due to the heterogeneity of the project format and an impending restructuring of the project formats, which meant that this project would be discontinued. The data are presented in Fig. 1, which shows that students received the least amount of feedback for the oral presentation, followed by the poster presentation and the patient information leaflet. These differences were statistically significant when analysed by the Kruskal-Wallis test (data are not normally distributed) with *post hoc* Dunn's multiple comparison tests. In summary, the amount of feedback provided for the oral presentation, poster presentation and patient information leaflet was significantly lower than that provided for the other two projects. There was no statistically significant difference among the oral presentation, poster presentation and patient information leaflet.

Feedback-form restructuring was implemented for all projects following the initial evaluation. The two projects receiving the lowest feedback were then chosen for detailed evaluation: the oral presentation and the poster presentation. The patient information leaflet was not included as this assignment was to be discontinued.

### Oral presentation

**Fig. 2:**
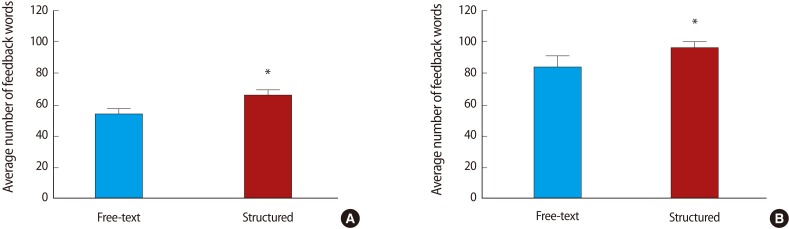
More feedback (mean number of words) was entered on the structured feedback form versus the free-text feedback form for two different assignments. (A) Average number of words per feedback event for oral presentations. (B) Average number of words per feedback event for poster presentations. Data compared by unpaired t-test. ^*^P<0.05.

**Fig. 3:**
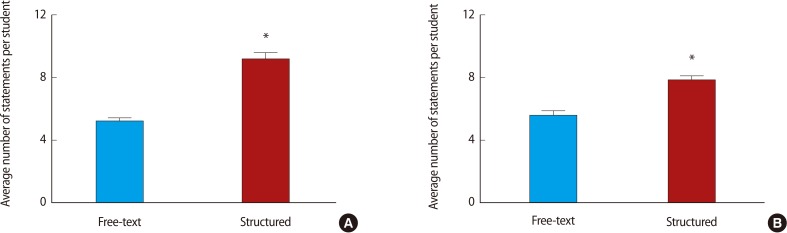
More feedback (mean number of statements) was entered on the structured feedback form vs. the free-text feedback form for two different assignments. (A) Average number of statements per feedback event for oral presentations. (B) Average number of statements per feedback event for poster presentations. Data compared by unpaired t-test. ^*^P<0.05.

**Fig. 4:**
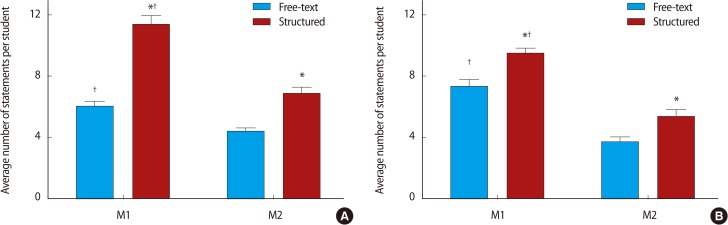
'First markers' write significantly more feedback than 'second markers' and this difference is consistent whether the feedback form is free-text or structured. (A) Average number of statements for marker 1 and marker 2 using the free-text and structured feedback forms. (A) Average number of statements for marker 1 and marker 2 using the free-text and structured feedback forms. Data compared by two-way ANOVA with *post hoc* Bonferroni test. ^*^P<0.05 when compared to free-text condition. ^†^P<0.05 when compared to marker 1.

**Fig. 5:**
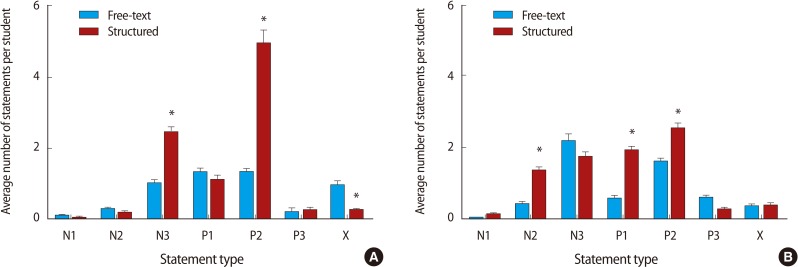
Use of a structured feedback form was associated with an increase in the depth of feedback. (A) A comparison of statement type quality using the free-text and structured form format for oral presentations assessment. (B) A comparison of statement type quality using the free-text and structured form format for poster presentations assessment. See Table 1 for classification of statement types. Data compared by two-way ANOVA with *post hoc* Bonferroni tests. ^*^P<0.05.

Restructuring the feedback form to the structured style proforma was associated with a significant increase in the number of words (Fig. 2A) and feedback statements provided by both markers (Fig. 3A). Breakdown of the number of statements provided by each of the marker pairs revealed that marker 1 provided more feedback statements than marker 2, that this difference was maintained after the intervention, and that form restructuring was associated with an increase in the number of statements provided by each of the markers (Fig. 4A). Classification of the feedback into the statement types demonstrated that much of the increase was due to statistically significant increases in 'N3' and 'P2' type statements and a decrease in neutral statements 'X' (Fig. 5A). It is noteworthy that, even before the intervention, there was very little feedback in categories 'N1' and 'X'; these two categories together contributed 19.9% of the total feedback before the intervention and only 2.9% after.

There was no statistical difference in the assessment mark awarded to students completing the assignment with the free-form or the structured proforma (blank: 73.05±7.943, restructured: 71.01±6.712, mean±SD; P>0.05 by unpaired t-test).

### Poster presentation

Restructuring the feedback form was associated with a significant increase in the number of words (Fig. 2B) and feedback statements provided by both markers (Fig. 3B). Breakdown of the number of statements provided by each of the markers revealed that marker 1 provided more feedback statements than marker 2, that this difference was maintained after the intervention and that form restructuring was associated with an increase in the number of statements provided by both markers (Fig. 4B). Classification of the feedback into the statement types demonstrated that much of the increase was accounted for by statistically significant increases in 'N2', 'P1', and 'P2' type statements (Fig. 5B). Again, very little feedback from categories 'N1' and 'X' was received either before or after the intervention; these two categories together contribute 6.1% of total poster feedback before and 5.6% after the change in feedback format.

As for the oral presentations, there was no statistical difference in the mark awarded to students completing the assignment with the 'free' form or the 'structured' proforma (blank: 67.99±7.353, restructured: 69.45±6.016, mean±SD; P>0.05 by unpaired t-test).

### Marker engagement and feedback on the intervention

In the 12 months since the intervention was initiated, no negative feedback has been received from markers regarding the form change, and there have been no late returns of marking and feedback. These elements suggest that the intervention has not adversely affected the workload for markers, although this has not yet been formally evaluated. Markers typically only entered text in two thirds of the domains available (63.4% of fields contained text for the oral presentation feedback, while the number for the poster presentation was 63.3%), suggesting that they did not feel it necessary to complete all 12 fields in the structured feedback proforma.

## DISCUSSION

This study aimed to address an ongoing issue in assessment by implementing an intervention designed to increase the quality and quantity of the feedback provided to students. An evaluation of this intervention revealed that, in two separate assignments, a change from a free-text feedback form to a structured feedback proforma was associated with an increase in both the amount of and quality of feedback provided to students. Additionally, it was determined that the vast majority of feedback provided using the new proforma was written in a way that could be useful for these transferable skills projects, with very little being of a purely negative (N1), neutral or irrelevant (X) nature. However, it was also found that first markers wrote more than second markers and that many markers still left sections blank on the structured feedback proforma. These data and analyses suggest that using a more structured feedback proforma could result in the provision of more abundant and detailed feedback to students.

Much of the literature on the provision of feedback in higher education concerns the feedback recipient - the learner. The needs of the educator are often overlooked. The provision of feedback is a complex instructional process that has many psychological considerations [16] and this intervention used very simple cue-questions to guide the educator. Further evaluations could explicitly examine the perceptions of the educators to determine if they felt the new proforma made it easier for them to deliver feedback. Although simple, the nature of the questions posed is very important. In a comprehensive review of educational feedback, Shute [1] indicates that high-achieving learners may benefit more from verification feedback, whereas low-achieving learners may benefit more from more directive, explicit feedback. The proforma described here facilitates the provision of both feedback types, due to the nature of the questions posed.

A number of important considerations need to be applied to interpret the apparent correlation between a change to the structured feedback form and the increase in the quantity and quality of feedback provided. Perhaps the most important is that it is not possible to conclude that the change from free-text to structured feedback forms has caused the increases in feedback quality and quantity. As is common with educational interventions, there are a number of variables which cannot be controlled for. Perhaps most significantly, feedback was provided to two different cohorts of students, 12 months apart. Nevertheless, the mean assignment mark awarded to the two cohorts was not statistically different, suggesting that the assignments being marked were of approximately equivalent standards. Also, the two groups of markers involved in the study are slightly different. Nevertheless, repeating the evaluation analyses and comparing only those markers who marked both free-text and structured feedback versions of each assignment produces approximately the same pattern of differences between the two feedback form approaches. These data are not shown for ease of presentation and also because they do not address the additional considerations (free-text and structured feedback forms used for different cohorts of students, projects 12 months apart). These issues are difficult to resolve, and it is for these reasons that we cannot conclude definitively that changing the form has caused the changes in the quantity and depth of the feedback provided. To do this might be possible with alternate approaches, such as requiring the same markers to assess the same group of assignments twice, once with the blank form and once with the structured form or using a control group. These strategies are problematic for a number of reasons, not least for the extra work required and the obvious telegraphing of the strategy being evaluated, which may prime markers and so influence the amount and quality of the feedback written. Using a control group would also potentially advantage or disadvantage some students. The current strategy was adopted as it did not disadvantage the students in any way, it required no additional work on behalf of the markers and it did not require any additional instruction to implement, thereby allowing our interpretation of the evaluation data to remain unclouded by any priming of the markers or the possibility of a placebo or Hawthorne effect wherein markers might provide different feedback simply because they were aware that their feedback was being evaluated.

Although the intervention was associated with an increase in the quantity and depth of the feedback, this does not absolutely guarantee that the feedback provided is actually more useful. The provision of feedback that can be clearly understood by students is a long-standing issue in higher education [8, 17] and a determination of whether the additional feedback provided to students is 'useful' or even welcomed would be difficult to determine conclusively, although it could form part of a future research study involving student input.

Finally, it was observed that, for these double-marked projects, the marker designated as 'marker 1' consistently provided more feedback than the marker designated as 'marker 2'. There are a number of possible explanations for this, but there exists the intriguing possibility that this effect results, in part, from the perceived importance of being designated 'marker 1' versus 'marker 2' and that this could be eliminated by removing the numerical designation. This is an area for future study.

An analysis of the literature on written feedback provision reveals an abundance of guidelines and training materials for teachers, with much of the research on feedback and its provision focused on ways to improve poor practice. Taking a faculty-wide approach, Mutch, writing in 2003 found that "most academics...were trying their best, in difficult circumstances, to give helpful feedback to their students" [9]. The evaluation described here demonstrates that, when classified according to a well-established and widely used tool, the majority of feedback provided to students was in fact of high quality, confirming Mutch's findings. Introducing a simple form restructuring approach such as that adopted here may further improve the quality and increase the amount of feedback provided. The study has demonstrated that this can occur without the need for resource-intensive training or a deficit approach where feedback is primarily focused on areas of poor performance.

In summary, changing from a free-text feedback form to a simple structured feedback proforma is associated with an increase in both the quantity and quality of feedback provided to graduate-entry medical students completing transferable skills projects at a UK University.

## Figures and Tables

**Table 1:**
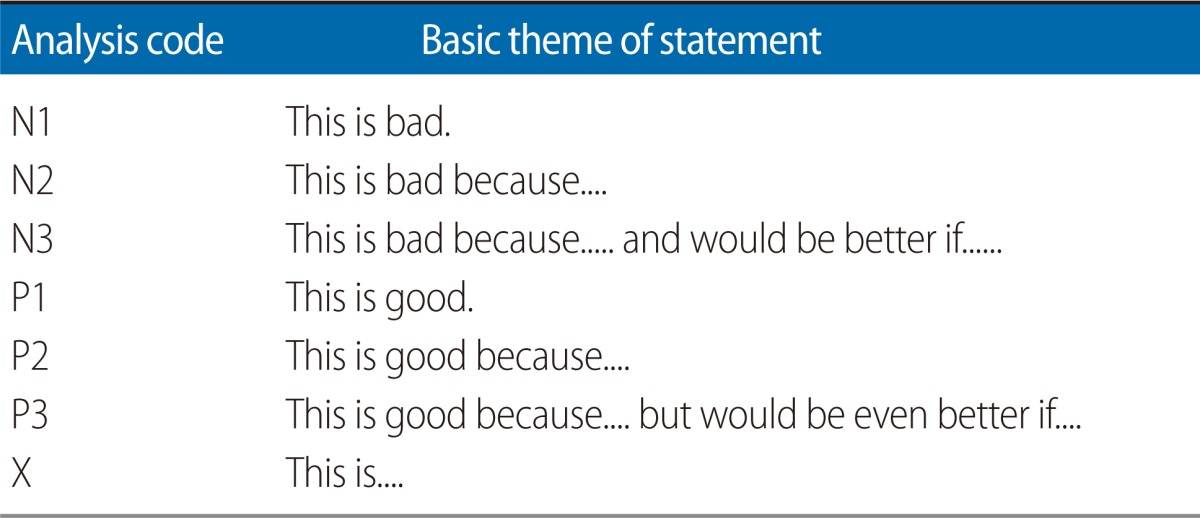
Classifications assigned to statements in feedback

Adapted from Glover and Brown [11].
